# Medical Department of the Royal Navy

**Published:** 1902-10-25

**Authors:** 


### The Institutional Workshop.

### MEDICAL DEPARTMENT OF THE ROYAL NAVY.

### REGULATIONS FOR THE ENTRY OF SURGEONS FOR TEMPORARY SERVICE.

The following are the recently established regulations for the entry of surgeons for temporary service in the Royal Navy :—

Surgeons who may be temporarily employed in the Royal Navy to meet the requirements of the service will be appointed under the following regulations :—

#### Qualifications.

To be registered under the Medical Act as qualified to practise medicine and surgery in Great Britain and Ireland.

To produce certificates of good character.

To be reported physically fit after medical examination.

Age not to exceed 40 years.

#### Pay and Allowances.

Full pay—a day, 22s. ; a year, £401 10s.

Half pay—a day, 10s. ; a year, £182 10s.

*Note.*—Half pay is for sickness and extra leave only.

To be granted 30 days' advance of pay on joining a ship after appointment.

To receive the same allowances as are payable to permanent officers of their rank.

Lodging money at the rate of £50 a year is usually allowed when employed on shore without quarters in the United Kingdom, and £24 a year in lieu of rations. In cases however of temporary employment on shore, the lodging and provision allowances will be at the rate of 3s. 6d. and 1s. 6d. per day respectively.

If quarters are provided in a medical establishment, an allowance is granted in lieu of provisions, for self and servant, and for fuel and lights at the rate of £39 a year in the United Kingdom, and £108 a year abroad.

#### Uniform.

Each surgeon to provide himself as follows :—

(1) Frock coat, waistcoat, and trousers.
(2) Undress coat.
(3) Uniform cap.
(4) Mess jacket and waistcoat.
(5) Sword and undress belt.

All as specified in the Uniform Regulations.

To cover the above each officer accepted for service will receive an equipment allowance of £20 (payable on the officer's being called up for active service).

The following instruments must be provided by the surgeon :—

A pocket case of instruments.
A stethoscope.
Three clinical thermometers.

#### Messing.

Surgeons will be allowed, when attached to ships in commission, the ordinary ship's rations ; but will have to pay about 2s. a day towards the maintenance of their mess as ward-room officers.

#### Pensions for Wounds and to Widows, etc.

In the event of surgeons engaged for temporary service being wounded in His Majesty's service, gratuities or pensions, varying in amount according to the injuries sustained, will be granted, on the basis of the awards in similar cases of Naval officers.

Should temporary service surgeons be killed in action, die within six months of wounds received in action, or meet their death by acts of the enemy, pensions and allowances will be granted to their widows, children, etc.

In addition to the foregoing pensions, the widows and children of officers killed in action will be granted the following gratuities :—

Widows—One year's pay of their husbands' corresponding rank in the Royal Navy.

Each unmarried child, under the age of 21, one-third of the gratuity paid to the widow.

These pensions and gratuities can be given only in cases of injury or death caused by acts of the enemy, and not on account of injury, disability, or death which may result from carrying on the ordinary duties of the service.

[Preference will be given to candidates who are unmarried.]

#### Conditions of Service.

To engage for six months certain, but the liability to serve will be limited to five years.

To serve when and where required from the date of signing the declaration.

To be liable to immediate discharge for misconduct or incompetency.

To rank with, but after, surgeons in the permanent service.

To be under the general rules of the service as regards discipline, etc.

To receive two calendar months' notice of services being no longer required.

To be granted a gratuity of two calendar months' pay on discharge, if not discharged for misconduct or incompetency.

Voluntary resignation of appointment will be allowed subject to the convenience of the service, but the gratuity of two calendar months' pay on discharge will be thereby forfeited.

N.B.—Copies of the Regulations and Declaration can be obtained from the Medical Department of the Navy, Admiralty, London, S.W.

